# A Small-Crystallite Metal–Organic Framework for Efficient CO_2_ Capture from Flue Gas

**DOI:** 10.3390/molecules31193500

**Published:** 2026-10-01

**Authors:** Ying Yuan, Chen Huang, Yu-Jia Chen, Xu Li, Hong-Wei Chen, Li-You Liang, De-Hua Lu, Min Liu, Ya-Min Wu, An-Min Zhao, Yi-Hong Wu, Yan-Xin Chen

**Affiliations:** 1 State Key Laboratory of Porous Materials for Separation and Conversion, Southwest Research & Design Institute of the Chemical Industry, Chengdu 610225, China; 2 State Key Laboratory of Structural Chemistry, Fujian Science & Technology Innovation Laboratory for Optoelectronic Information of China, and Fujian Institute of Research on the Structure of Matter, Chinese Academy of Sciences, Fuzhou 361021, China

**Keywords:** MOF modulation, morphology modulation, CO_2_ capture, adsorption kinetics

## Abstract

Pressure swing adsorption (PSA) is a promising technology for low-energy CO_2_ capture from flue gas, but its development is hindered by the lack of advanced adsorbents. Aluminum formate (ALF) shows satisfactory CO_2_ adsorption capacity but suffers from slow adsorption kinetics. Herein, we present a citric acid modification strategy to synthesize SNALF—an ultramicroporous ALF with small crystallite size and abundant defect sites—using nano-sized boehmite as the aluminum source. SNALF-3-90 achieved a CO_2_ adsorption capacity of 2.45 mmol/g within 3 min. In PSA testing with simulated flue gas (11 vol.% CO_2_, 82.5 vol.% N_2_, 6.5 vol.% O_2_), the CO_2_ concentration was enriched from 11% to over 42% with 90.13% recovery and productivity of 0.80 NL/min. DFT calculations reveal that the ligand-defective SNALF surface enables strong exothermic adsorption, confirming that the introduced defects act as active centers for CO_2_ capture. This work demonstrates that defect engineering combined with crystallite size reduction significantly enhances the CO_2_ adsorption kinetics of ALF, making it viable for large-scale PSA-based flue gas capture.

## 1. Introduction

The combustion of fossil fuels has resulted in substantial CO_2_ emissions, driving the urgent need for efficient CO_2_ capture technologies [1,2,3]. Current approaches include absorption, adsorption, membrane separation, cryogenic distillation, and electroswing adsorption [4]. Among these, adsorption is widely employed in industry owing to its energy efficiency, high automation, and environmental friendliness [5,6,7], where the adsorbent is the key determinant of separation performance and process economics. Metal–organic frameworks (MOFs) are crystalline porous materials constructed from metal nodes and organic bridging ligands, whose pore size and functionality can be flexibly tuned by varying the organic ligand, functional group, metal ion, and activation method [8,9,10]. With large specific surface areas and tunable porous structures, MOFs are promising adsorbents for high-efficiency CO_2_ capture [11].

So far, MOFs have demonstrated remarkable potential for CO_2_ capture [12,13,14,15,16,17,18]. However, most MOFs have their own limitation such as poor stability, high raw material costs and poor moisture resistance. Aluminum-based MOFs (Al-MOFs) have attracted intensive attention owing to their low cost, high physicochemical stability, and diverse structures and functions [19]. Li et al. [20] reported that a series of Al-MOFs assembled from Al^3+^ and terephthalate anions exhibited high stability and CO_2_ selectivity, and that CO_2_ and water adsorption behavior could be fine-tuned by tailoring the internal surface environment. As the simplest metal formate, ALF exhibits excellent CO_2_/N_2_ separation performance for flue gas, attributable to its unique hydrogen-confined pore cavities—where inward—pointing formate hydrogens form hydrogen bonds with CO_2_, promoting strong guest–host interactions [21,22]. In a recent study, iron was introduced into the ALF network to optimize the pore environment and gas diffusion pathways, and polyvinylidene fluoride was further employed for moisture-resistance passivation (Fe-ALF-PVDF) [23]. Despite these advantages, the excessively small pore size of ALF enhances molecule–pore wall interactions and induces steep temperature gradients during adsorption, thereby limiting gas diffusion and reducing the adsorption kinetics [24]. Notably, reducing the crystallite size is an effective strategy to enhance the adsorption kinetics [25]. Indeed, nanoscale Al-MOFs have been reported in various contexts: aluminium fumarate (AlFu) nanoparticles (20–30 nm) as dispersive adsorbents [26], nano AlFu decorated on diatoms for rapid dye adsorption [27], and ~50 nm AlFu crystals embedded in aerogels with accelerated ion diffusion [28]. Related Al-Fum-Al_2_O_3_ composites have also been studied for water adsorption and thermochemical heat storage [29]. However, these precedents concern fumarate-based Al-MOFs in liquid-phase or water adsorption rather than CO_2_ capture, and nano-AlFu is prone to agglomeration in aqueous media [30]. The relationship between crystallite-size reduction and enhanced CO_2_ adsorption kinetics has not previously been established for ALF, whose hydrogen-bond-confined pore cavities give rise to distinct adsorption and diffusion behavior.

Herein, we propose a novel SNALF with substantially reduced crystallite size through the synergistic combination of raw material optimization and citric acid modification. Two core modification mechanisms are elaborated as follows. First, boehmite nano-Al(OH)_3_ replaces conventional gibbsite Al(OH)_3_ as the aluminum source. Nano-sized boehmite possesses superior ligand coordination activity, which accelerates nucleation and restricts crystal growth into smaller crystallite. Citric acid acts as a modulator that competes with formic acid for Al coordination during crystallization. After the subsequent washing and drying processes, the weakly bound citric acid is removed, leaving behind permanent ligand vacancies (missing formate defects) and an irregular framework structure that enhance CO_2_ affinity. Compared with pristine ALF, the as-synthesized SNALF exhibits significantly reduced crystallite size, faster adsorption kinetics, and superior CO_2_ adsorption performance. By systematically optimizing the structure of ALF via the dual strategy, we successfully overcome its kinetic limitation, providing a scalable adsorbent candidate for industrial PSA flue gas CO_2_ separation.

## 2. Results and Discussion

### 2.1. Structural and Textual Properties of Samples

**Figure 1:**
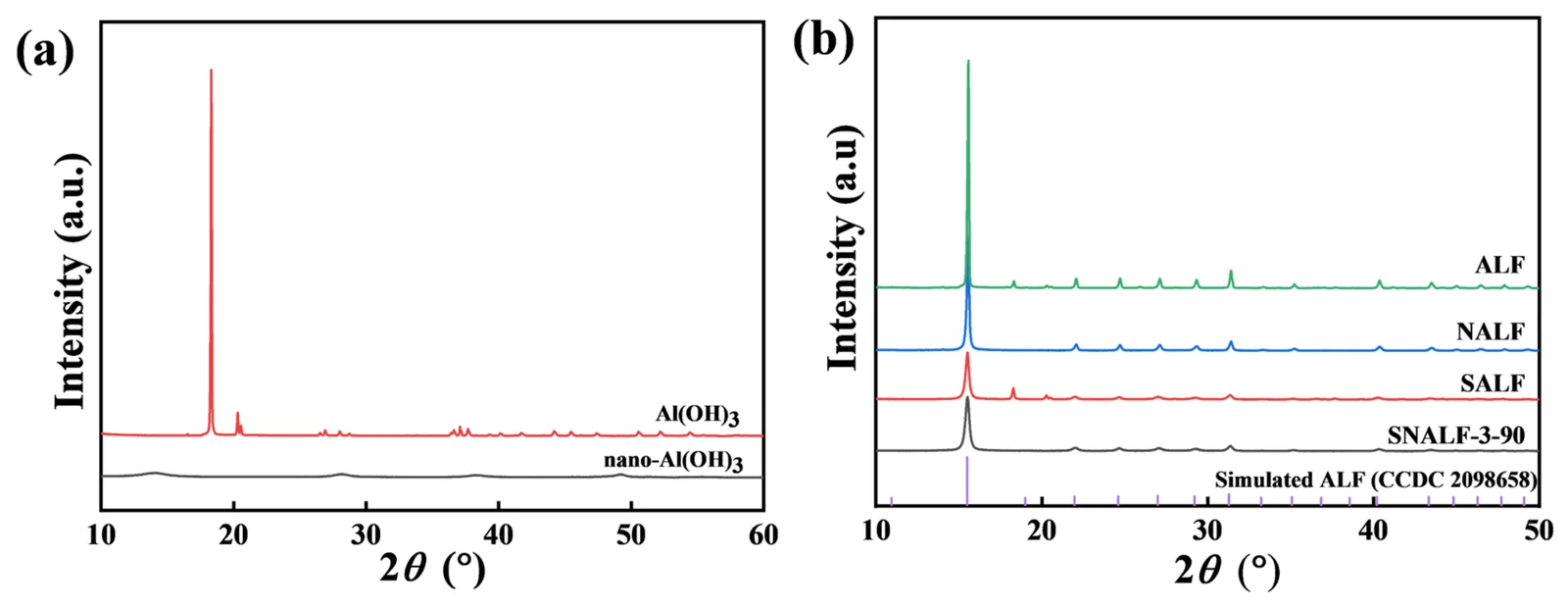
XRD patterns of Al(OH)_3_ and nano-Al(OH)_3_ (**a**), ALF, NALF, SALF, SALF-3-90 (**b**) and the simulated PXRD pattern of ALF.

The crystal structure of as-prepared samples was explored by XRD characterization; the XRD patterns are presented in Figure 1. As shown in Figure 1a, the diffraction peaks are observed at 14.5°, 28.2°, 38.4°, and 49.0°, for nano-Al(OH)_3_ correspond to the boehmite (PDF 05-0190), whereas the peaks at 18.3° and 20.4° for Al(OH)_3_ are assigned to the gibbsite (PDF 74-1775). Notably, the diffraction peak intensity of Al(OH)_3_ is considerably higher than that of nano-Al(OH)_3_, indicating the lower crystallinity and smaller crystallite size of the latter. As depicted in Figure 1b, the principal characteristic diffraction peaks of ALF, NALF, SALF, and SNALF-3-90 are in good agreement with both the simulated XRD pattern of ALF calculated from the single-crystal structure and the patterns of aluminum formate reported in the literature [21,22,31], demonstrating that neither the substitution of nano-Al(OH)_3_ for Al(OH)_3_ as the aluminum source nor citric acid modulator alters the original framework structure. Interestingly, the diffraction peaks of NALF exhibit weaker intensities compared with those of ALF, with the characteristic peak of unreacted Al(OH)_3_ at 2θ = 18.3° being absent. This phenomenon may be ascribed to the lower O-H bond strength at the terminal sites of nano-Al(OH)_3_ relative to that of Al(OH)_3_, the higher surface energy of the (001) and (100) crystal planes of boehmite compared with those of gibbsite, and the enhanced reactivity of boehmite. These factors collectively facilitate the adsorption and coordination with ligand molecules, as well as promoting rapid and extensive nucleation during the initial stage of the reaction, thereby yielding a greater number of smaller crystallites [32]. It is also noteworthy that the citric acid-modified samples (SALF and SNALF-3-90) exhibit a marked decrease in characteristic peak intensities. This may be attributed to the competitive coordination between citric acid and formic acid for aluminum ions in solution, which generates defect sites and induces the formation of an irregular framework structure [33,34].

**Figure 2:**
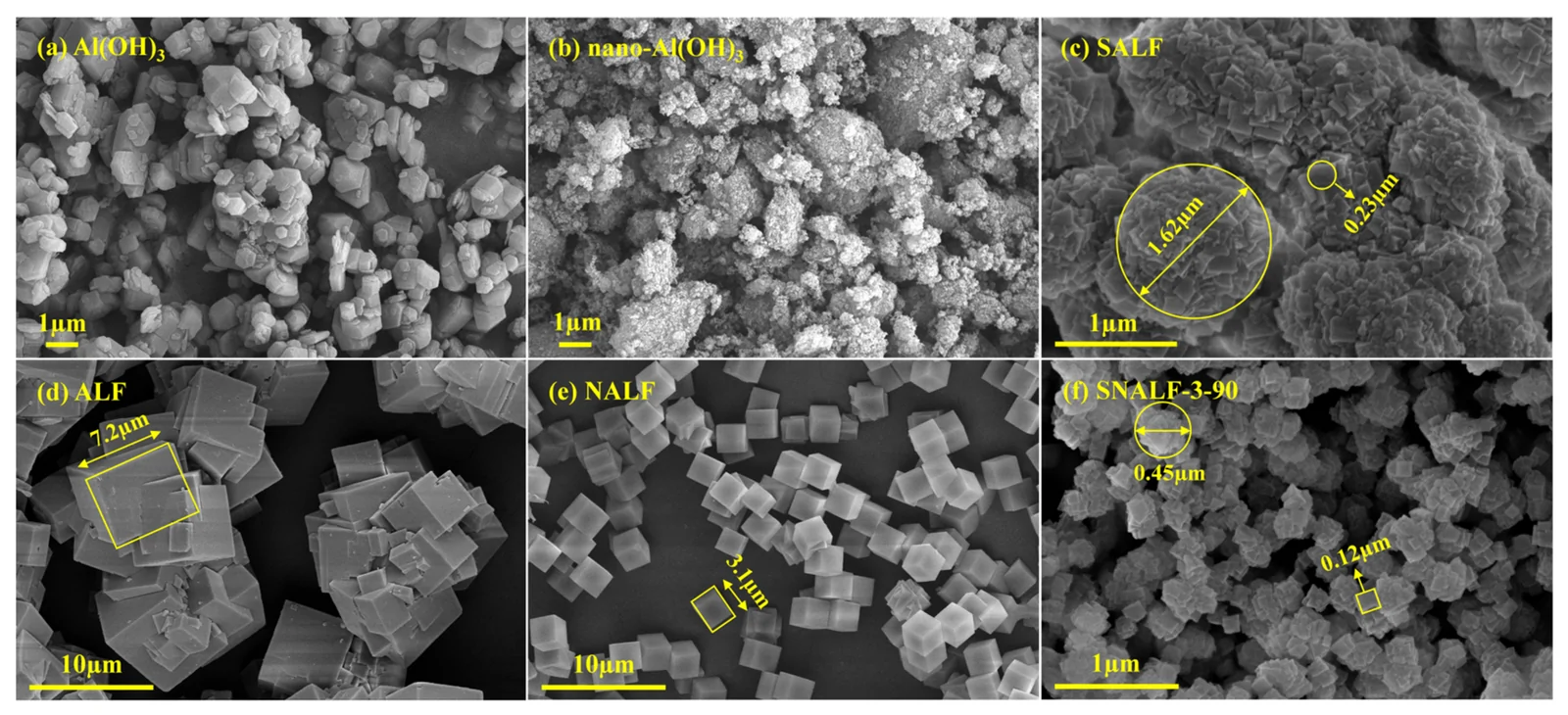
SEM images of Al(OH)_3_ (**a**), nano- Al(OH)_3_ (**b**), SALF (**c**), ALF (**d**), NALF (**e**), and SNALF-3-90 (**f**).

To intuitively elucidate how aluminum source type and citric acid modification regulate sample micromorphology, SEM characterization was performed on the Al(OH)_3_ and nano-Al(OH)_3_ aluminum sources, as well as the ALF, NALF, SALF, and SNALF-3-90 samples, as shown in Figure 2. A comparison of Figure 2a,b reveals that nano-Al(OH)_3_ exhibits a more loosely packed structure with smaller crystallites, which is more favorable for the coordination between aluminum ions and organic ligands. As shown in Figure 2d,e, ALF exhibits irregular cubic crystallites with severe agglomeration, whereas NALF exhibits well-dispersed regular cubic crystallites with a particle-size reduction of approximately 4.1 µm. A further comparison of SALF and SNALF-3-90, prepared from different aluminum sources with citric acid modification, reveals that SNALF-3-90 exhibits improved dispersibility and smaller crystallites, with the size of aggregated spherical particles reduced by approximately 1.17 µm. The corresponding size distribution is provided in Figure S1. The counting statistics support the qualitative SEM observations that NALF has a smaller average particle size than ALF and that citric acid modified SNALF-3-90 exhibits a much narrower size distribution. This further confirms that the substitution of Al(OH)_3_ with nano-Al(OH)_3_ in the synthesis process can reduce crystallite size and enhance dispersibility, thereby significantly improving the kinetic diffusion rate [25]. Furthermore, a comparison of Figure 2c–f demonstrates that the morphology of citric acid-modified samples undergoes significant changes, accompanied by a substantial reduction in crystallite size. This indicates that citric acid modification effectively inhibits crystallite growth, resulting in abundant irregular small-crystallite structures, which is consistent with the XRD characterization results.

**Figure 3:**
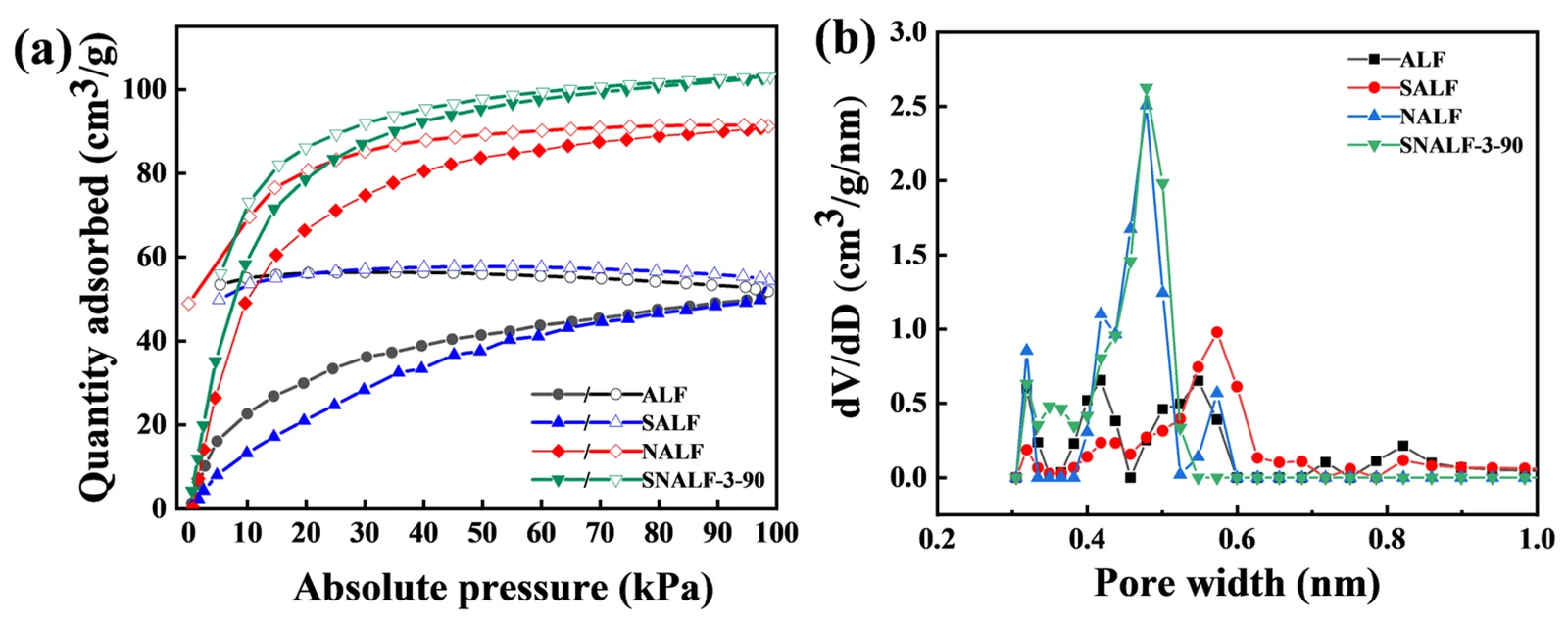
(**a**) CO_2_ adsorption/desorption isotherm at 273 K and (**b**) the pore size distribution of ALF, SALF, NALF and SNALF-3-90.

To investigate the pore characteristics of ALF, NALF, SALF, and SNALF-3-90, CO_2_ adsorption–desorption isotherms were measured at 273 K (Figure 3a). All samples exhibit Type I isotherms according to the IUPAC classification, featuring a steep uptake at low pressure followed by a plateau without capillary condensation, confirming their microporous nature. Notably, NALF and SNALF-3-90 display substantially higher CO_2_ uptake than ALF and SALF, indicating that the use of nano-Al(OH)_3_ as the aluminum source generates abundant CO_2_-accessible ultramicropores (Table 1). The desorption branch does not fully retrace the adsorption branch, giving rise to a low-pressure hysteresis commonly observed in ultramicroporous materials [35]. This is attributed to the kinetic trapping of CO_2_ molecules within the ultramicropores.

The pore size distribution (PSD) curves obtained by NLDFT fitting of the CO_2_ (273 K) adsorption isotherms using a slit-pore carbon kernel are presented in Figure 3b, which verify the ultramicroporous texture of all samples. For NALF and SNALF-3-90 prepared with nano-Al(OH)_3_, a relatively intense peak centered primarily in the range of 0.38–0.53 nm is observed, indicating a more ordered crystal structure with uniform pore size distributions, in good agreement with the FESEM observations. It can also be observed from Figure 3b that the significantly higher peak intensity in the range of 0.45–0.62 nm for SALF compared to ALF suggests that citric acid enlarges the pore size. The reported crystal structure of ALF (Al(HCOO)_3_) comprises small (SC) and large (LC) cages with channels distances of 4.10–5.29 Å [21]. These crystallographic dimensions are in agreement with the primary peak observed in the CO_2_-NLDFT PSD.

N_2_ adsorption–desorption measurements at 77 K were additionally performed for all samples (Table S1 and Figure S2). All samples exhibit low N_2_-BET surface areas, and the steep uptakes at high relative pressure indicate contributions from external surfaces and interparticle voids rather than framework micropores. Notably, the N_2_-BET values do not follow the same trend as the CO_2_ adsorption capacities, because the narrow ultramicropores (0.38–0.53 nm) are kinetically inaccessible to N_2_ at 77 K, whereas they are effectively probed by CO_2_ at 273 K [36]. This apparent inconsistency arises from the different accessible pore regimes probed by the two gases under their respective measurement conditions.

**Figure 4:**
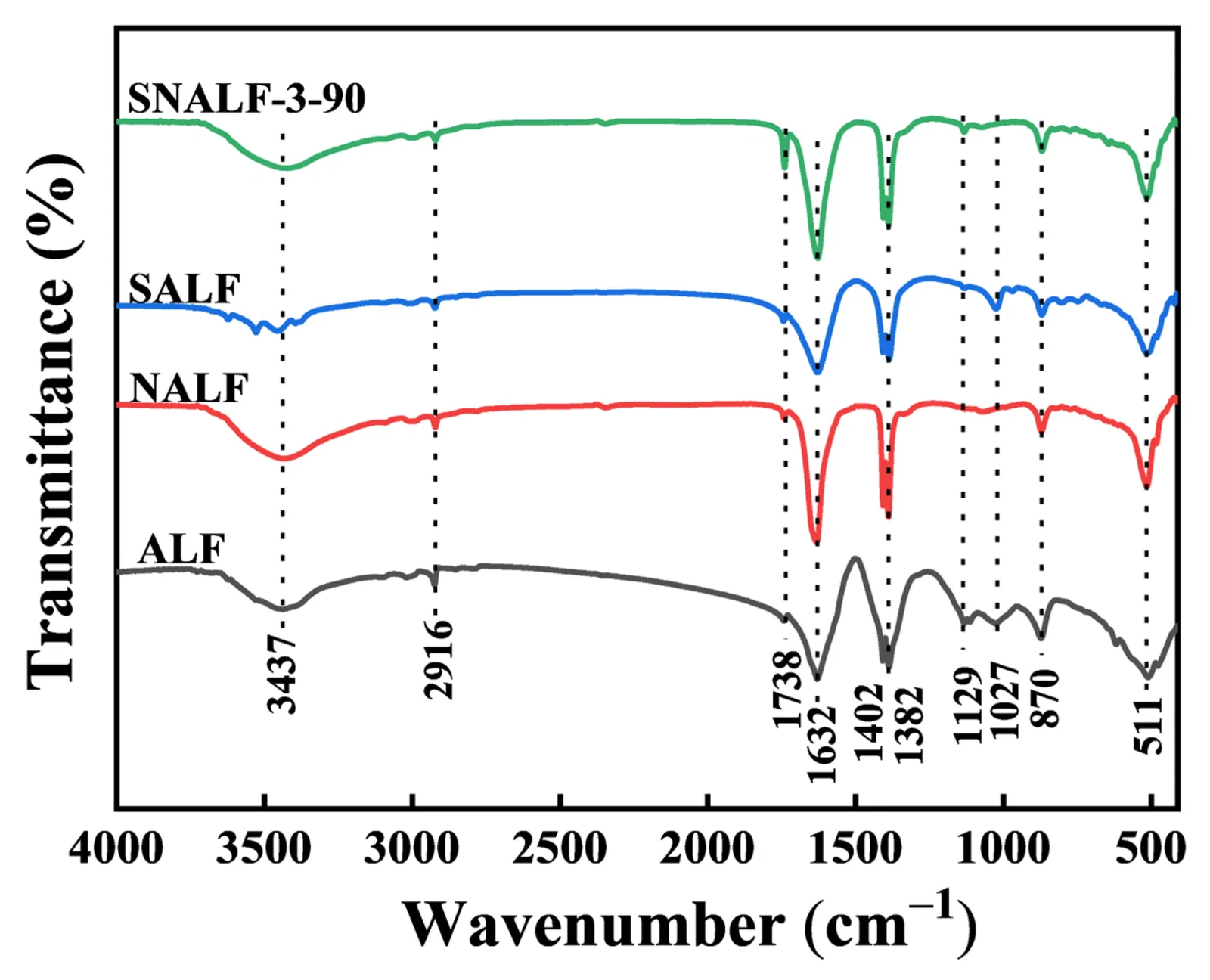
FT-IR spectra of ALF, NALF, SALF and SNALF-3-90.

FTIR spectra of ALF, NALF, SALF and SNALF-3-90 samples are depicted in Figure 4. The wide peak at 3437 cm^−1^, observed for all samples, is assigned to the O–H stretching vibration of surface-adsorbed water. The vibration bands at 1632 cm^−1^, 1402 cm^−1^, 1382 cm^−1^ and 870 cm^−1^ are assigned to the C=O vibration, respectively representing the stretching, symmetric, asymmetric and deformation vibration. The peaks at 2918 cm^−1^, 1129 cm^−1^ and 511 cm^−1^ are the C-H deformation vibration. The aforementioned vibrational bands are characteristic of a bidentate bridging coordination mode between aluminum ions and carboxyl group [26]. Notably, a distinct peak appears at 1738 cm^−1^ in the spectra of SALF and SNALF-3-90, which is significantly weaker or absent in the ALF and NALF. This peak is assigned to the C=O stretching vibration of protonated (uncoordinated) carboxylic acid groups [37]. This is likely attributed to the competitive coordination between citric acid and formic acid, which impedes the complete cross-linking of the framework and consequently leads to the formation of ligand-defective sites terminated by protonated carboxyls [38]. As shown in Figure 4, the peak of the C=O stretching vibration of NALF and SNALF-3-90 at 1632 cm^−1^ becomes stronger. This might be due to the stronger covalent bond interaction between the boehmite aluminum ions and the carboxyl group, resulting in a stronger delocalization of electrons on the two oxygens atoms [39]. 

**Figure 5:**
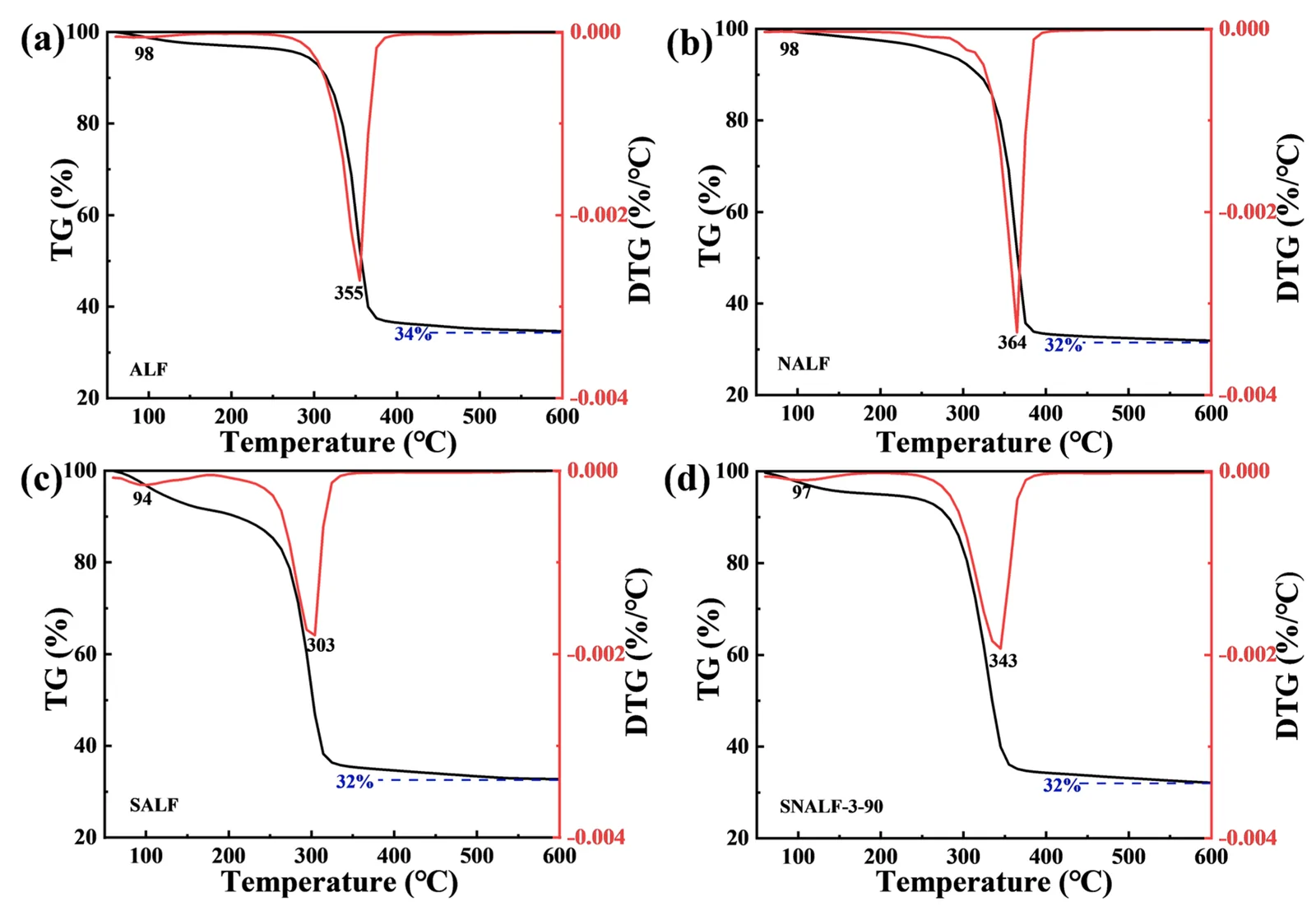
TG and DTG curves of ALF (**a**), NALF (**b**), SALF (**c**), and SNALF-3-90 (**d**) under Ar stream.

Thermogravimetric-differential (TG-DTG) analyses were performed on ALF, NALF, SALF, and SNALF-3-90 under Ar atmosphere in Figure 5. The thermal degradation process of these samples can be divided into two stages according to the DTG peaks. In the first stage below 150 °C, the minor weight loss is attributed to the evaporation of adsorbed solvents. The second stage, characterized by the major DTG peaks, corresponds to the decomposition of ligands and the collapse of the framework structure. Notably, the framework collapse temperature decreases from 355 °C for ALF to 303 °C for SALF, indicating that the use of citric acid during synthesis significantly compromises the thermal stability of the resulting framework. This may be ascribed to the high density of formate vacancies generated by the modulator, which interrupt the Al–O–C coordination network and weaken the local Al–O bond strength [5,40]. In contrast, the use of nano-Al(OH)_3_ as the aluminum source partially mitigates this destabilization effect, as evidenced by the higher collapse temperature of SNALF-3-90 (343 °C) compared to that of SALF, indicating that SNALF-3-90 possesses superior thermal stability. The final residual mass of approximately 32–34% corresponds to the formation of alumina [21].

With respect to a quantitative analysis of the role of citric acid, to unambiguously distinguish between framework incorporation, residual adsorption, and modulator behavior of citric acid, we combined digestion-based quantitative ^1^H NMR with thermogravimetric analysis under oxidative conditions. ^1^H NMR of the digested material (Figure S3) shows a pronounced formate resonance (8.24 ppm; 53.2 wt%), whereas no citrate-derived resonances (2.54–2.45 ppm) are observed above the quantification limit, corresponding to ≤0.02 wt% citric acid and an FA:CA molar ratio of at least 2 × 10^4^:1. The constant Al_2_O_3_ residue above 600 °C (31.7 wt%) corresponds to 6.22 mmol Al per gram of material (Figure S4); combined with the NMR data, this yields a framework FA/Al ratio of 1.90. Notably, the calculated Al_2_O_3_ residue of 31.70 wt% is in exact agreement with the experimental TGA value. We therefore conclude that citric acid is not incorporated into the framework of SNALF and is removed by the washing and drying processes; instead, it acts as a modulator that generates ligand-vacancy defect.

### 2.2. Adsorption Performance of Samples

**Figure 6:**
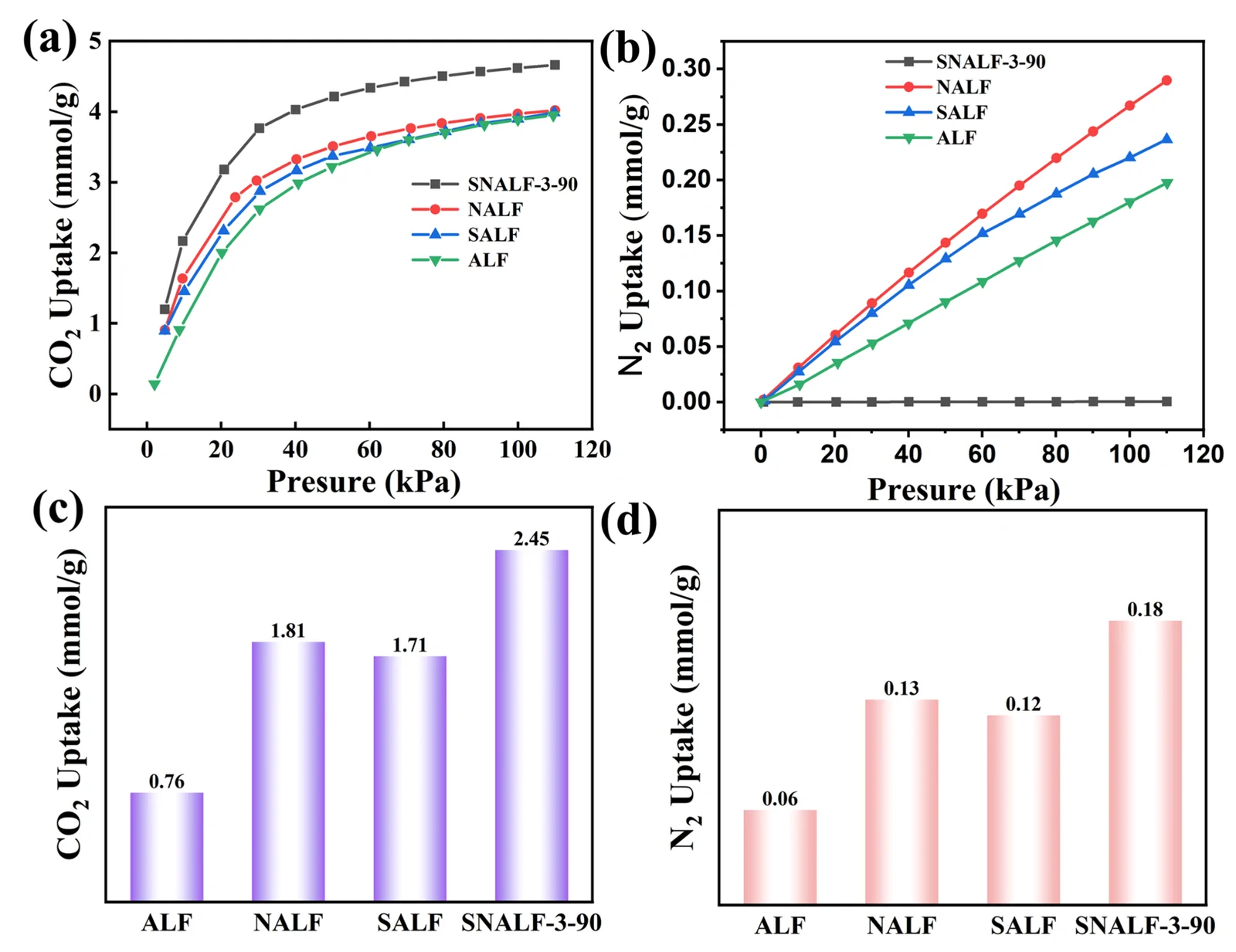
(**a**) The equilibrium CO_2_ adsorption capacity, (**b**) the equilibrium N_2_ adsorption capacity, (**c**) static CO_2_ adsorption capacity within 3 min, and (**d**) static N_2_ adsorption capacity within 3 min of ALF, SALF, NALF and SNALF-3-90.

While equilibrium adsorption capacity reflects the theoretical upper limit of adsorbent adsorption capability, it offers limited guidance for real industrial PSA applications. PSA processes are inherently constrained by efficiency and energy consumption considerations, with typical cycle adsorption times not exceeding 3 min, whereas achieving full adsorption equilibrium often requires several hours or even longer. Consequently, evaluating adsorption performance based on CO_2_ adsorption capacity within 3 min—termed static adsorption capacity—provides a more industrially relevant metric. The equilibrium and static adsorption capacities of the synthesized samples are presented in Figure 6. Both samples prepared via aluminum source modification and citric acid treatment demonstrate markedly enhanced adsorption performance compared to pristine ALF. Notably, SNALF-3-90, which was subjected to simultaneous modification by both aforementioned strategies, exhibited optimal performance, achieving equilibrium and static CO_2_ adsorption capacities of 4.66 mmol/g and 2.45 mmol/g, respectively. Based on static adsorption capacities within 3 min, the dynamic CO_2_/N_2_ selectivity for SNALF-3-90 reaches approximately 13.6, higher than that of pristine ALF. These results, in conjunction with the aforementioned characterization data, indicate that nano-Al(OH)_3_ and citric acid exert a synergistic effect. Specifically, the combined modification strategy effectively reduces crystallite size, and generates abundant coordination defect active sites. Such structural advantages accelerate mass transfer kinetics and drastically boost the overall CO_2_ adsorption capacity of the adsorbent.

### 2.3. Effects of Other Factors on the Structure and Adsorption Performance of Adsorbents

To fully elucidate the formation mechanism of SNALF adsorbents, systematic controlled experiments were carried out to clarify how citric acid dosage and reaction temperature modulate crystal structures. All variable experimental conditions are summarized in Table 2.

**Figure 7:**
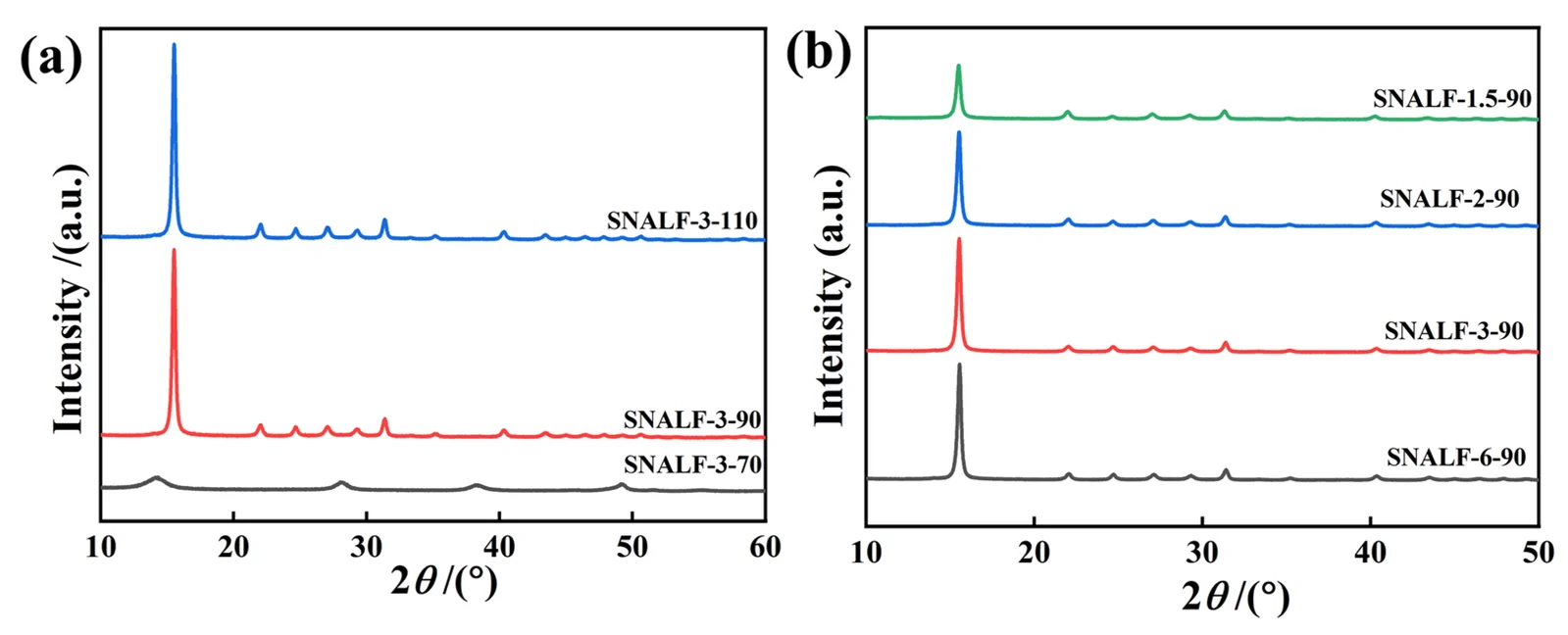
XRD patterns of SNALF samples: (**a**) SNALF synthesized varying reaction temperatures; (**b**) SNALF synthesized with varying citric acid dosages.

The XRD patterns of samples synthesized with varying citric acid dosages and reaction temperatures are presented in Figure 7. The influence of reaction temperature on the crystal structure is illustrated in Figure 7a. The characteristic diffraction peaks of SNALF-3-90 and SNALF-3-110 are essentially identical, demonstrating that Al(HCOO)_3_ crystals can be successfully synthesized at both 90 °C and 110 °C. In contrast, the sample prepared at 70 °C exhibits characteristic diffraction peaks corresponding to the boehmite structure. This is presumably ascribed to the insufficient thermal energy at lower temperatures, which attenuates molecular motion and decreases the probability of effective collisions between reactants, thereby impeding the coordination between metal ions and ligands and ultimately preventing the nucleation of the target crystalline phase [41,42]. As depicted in Figure 7b, the diffraction peak intensity progressively diminishes with increasing citric acid dosage. This phenomenon can be attributed to the intensified competitive coordination between citric acid and formic acid, which suppresses crystal growth and consequently reduces the crystallite size [34].

**Figure 8:**
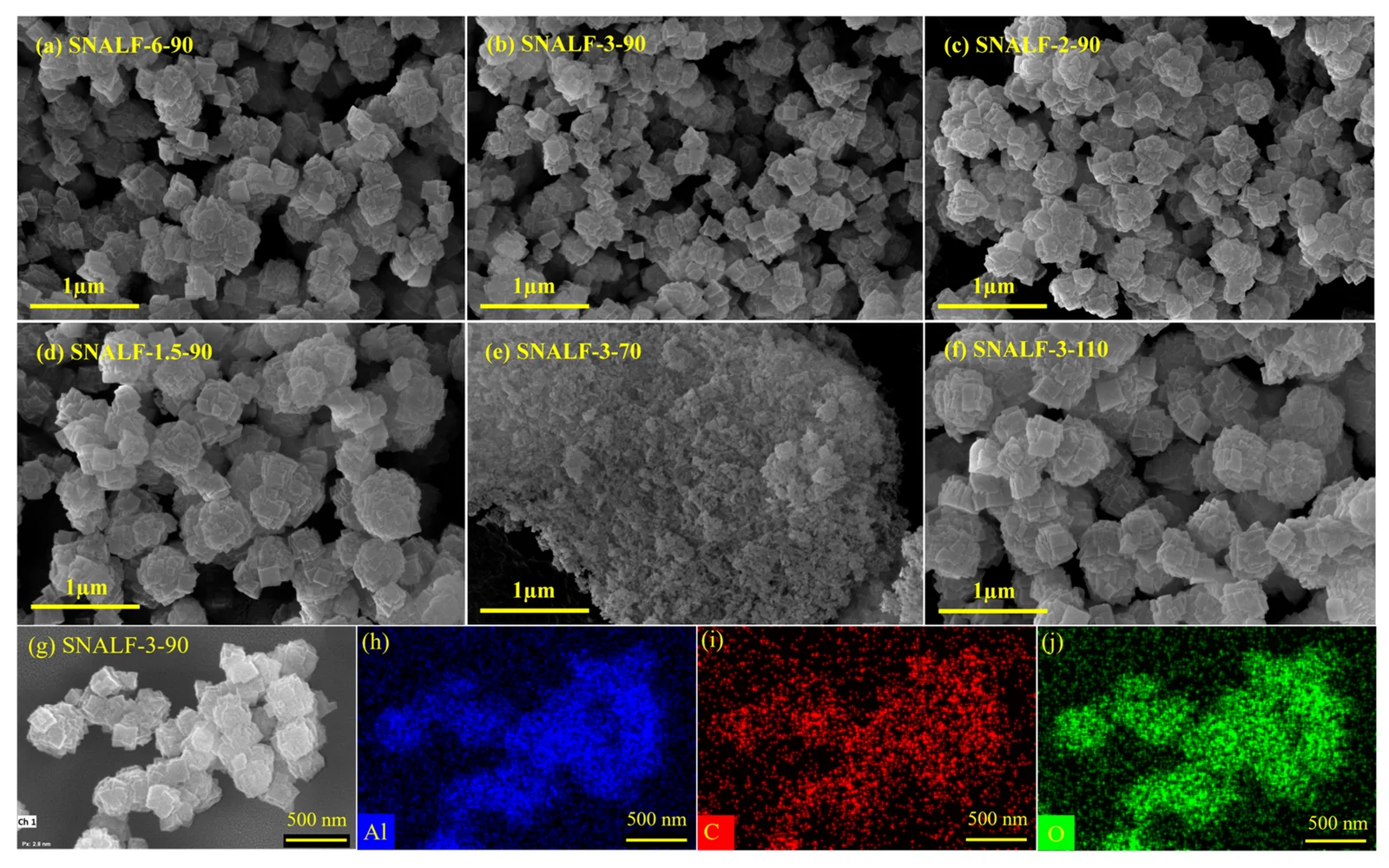
(**a**–**f**) SEM images of SNALF with varying citric acid dosages and reaction temperatures; (**g**–**j**) EDS mapping of SNALF-3-90: (**h**) Al (blue), (**i**) C (red), and (**j**) O (green).

The surface morphology of samples synthesized with different citric acid dosages and reaction temperatures was revealed by FE-SEM. As revealed in Figure 8a–d, the crystallite size decreased when the citric acid dosage was increased from 2 g (SNALF-6-90) to 4 g (SNALF-3-90). However, upon further increasing the citric acid dosage to 6 g (SNALF-2-90) and 8 g (SNALF-1.5-90), the crystallite size increased, accompanied by aggravated agglomeration. This phenomenon can be attributed to the excessive citric acid introducing abundant coordination defects inside the crystal lattice. These defect sites possess relatively lower energy, thereby accelerating crystal growth and resulting in enlarged crystallite sizes and intensified agglomeration [43]. The effect of reaction temperature on the morphology of samples is shown in Figure 8b,e,f. The morphology of SNALF-3-70 is similar to that of the nano-Al(OH)_3_, which is consistent with the XRD characterization results. A comparison between Figure 8b,f reveals that SNALF-3-90 synthesized at 90 °C exhibits a smaller crystallite size and superior particle dispersibility. To confirm the elemental composition and spatial distribution within the SNALF-3-90 sample, EDS mapping analysis was performed (Figure 8h–j). The results reveal that Al, C, and O elements are homogeneously distributed across the entire sample surface, indicating a uniform incorporation of these elements into the framework.

**Figure 9:**
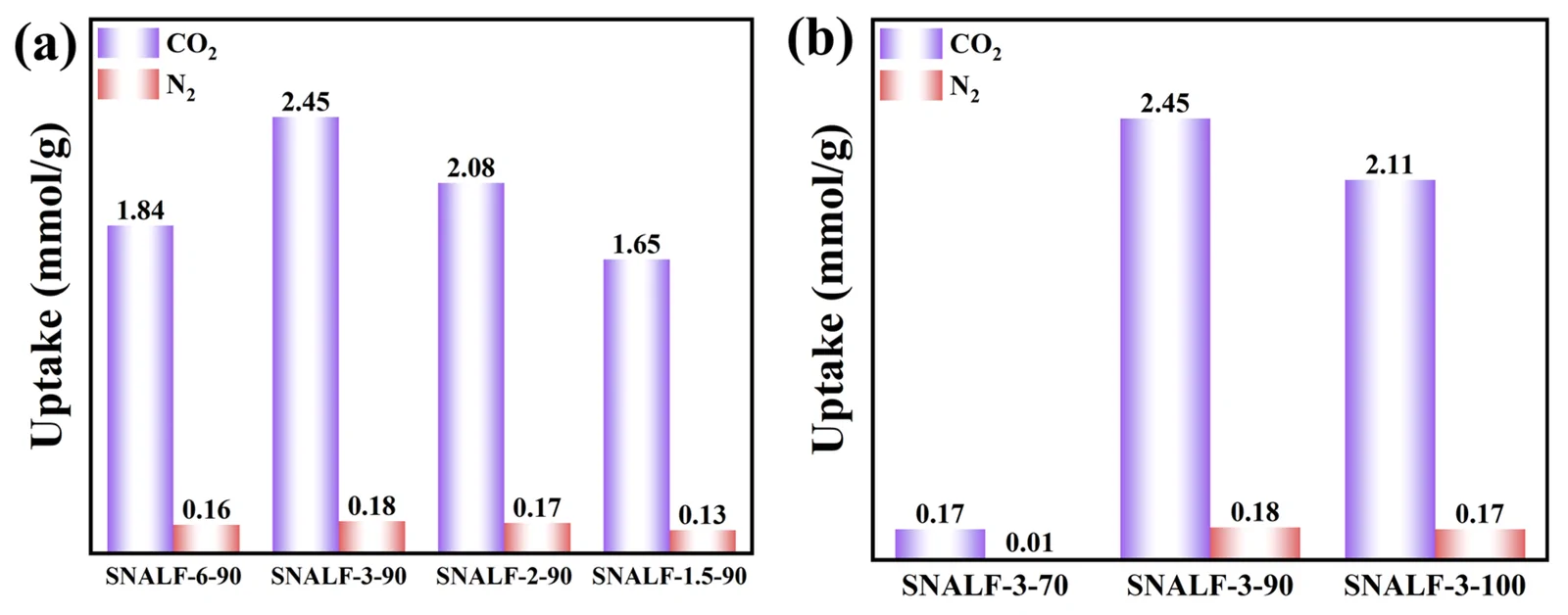
(**a**) Static adsorption results of synthetic samples at different additional dosages of citric acid; (**b**) static adsorption results of synthetic samples at different reaction temperatures.

The adsorption performance of the samples with different dosages of citric acid and reaction temperature was studied as shown in Figure 9. It can be seen from Figure 9a that the CO_2_ and N_2_ adsorption capacity of the adsorbent initially increases and subsequently decreases with increasing citric acid dosage, among which SNALF-3-90 demonstrates the optimal performance. Combined XRD and SEM characterization analyses reveal that excessive citric acid addition significantly reduces the crystallinity of the crystals and the dispersity of the crystallites, both of which are detrimental to the enhancement of adsorption performance. Compared to the dosage of formic acid, the additional dosage of citric acid is minimal, but it has significantly changed the crystal structure, surface morphology, thermal stability and adsorption properties of the material. This indicates the feasibility of using citric acid as a modifier to introduce vacancy defects into the crystal structure, thereby reducing the crystallite size. As shown in Figure 9b, with the increase in reaction temperature, the CO_2_ adsorption performance of the adsorbent first increases and then decreases. XRD characterization results demonstrate that Al(HCOO)_3_ crystals can be synthesized at both 90 °C and 110 °C. However, SEM results reveal that the sample synthesized at 90 °C exhibits a smaller crystallite size and superior particle dispersity, indicating that reducing crystallite size and enhancing dispersity can improve the CO_2_ adsorption performance of the sample.

To further clarify the origin of the adsorption performance, N_2_ adsorption–desorption measurements at 77 K were performed for all citric acid-modulated samples, and the results are summarized in Figure S5 and Table S2. All samples exhibit low N_2_-BET surface area, indicating that N_2_ probes mainly the external surfaces and interparticle voids rather than the framework ultramicropores. Notably, the CO_2_ uptake does not correlate with the N_2_-BET surface area or the total pore volume. This is consistent with the fact that the ultramicropores (0.38–0.53 nm) governing CO_2_ adsorption are kinetically inaccessible to N_2_ [36]. The optimal performance of SNALF-3-90 arises from the combined effect of a high ultramicropore fraction and the maximized accessibility thereof, enabled by its smaller crystallite size and superior dispersity.

### 2.4. Performance Evaluation of the Adsorbent by PSA

**Figure 10:**
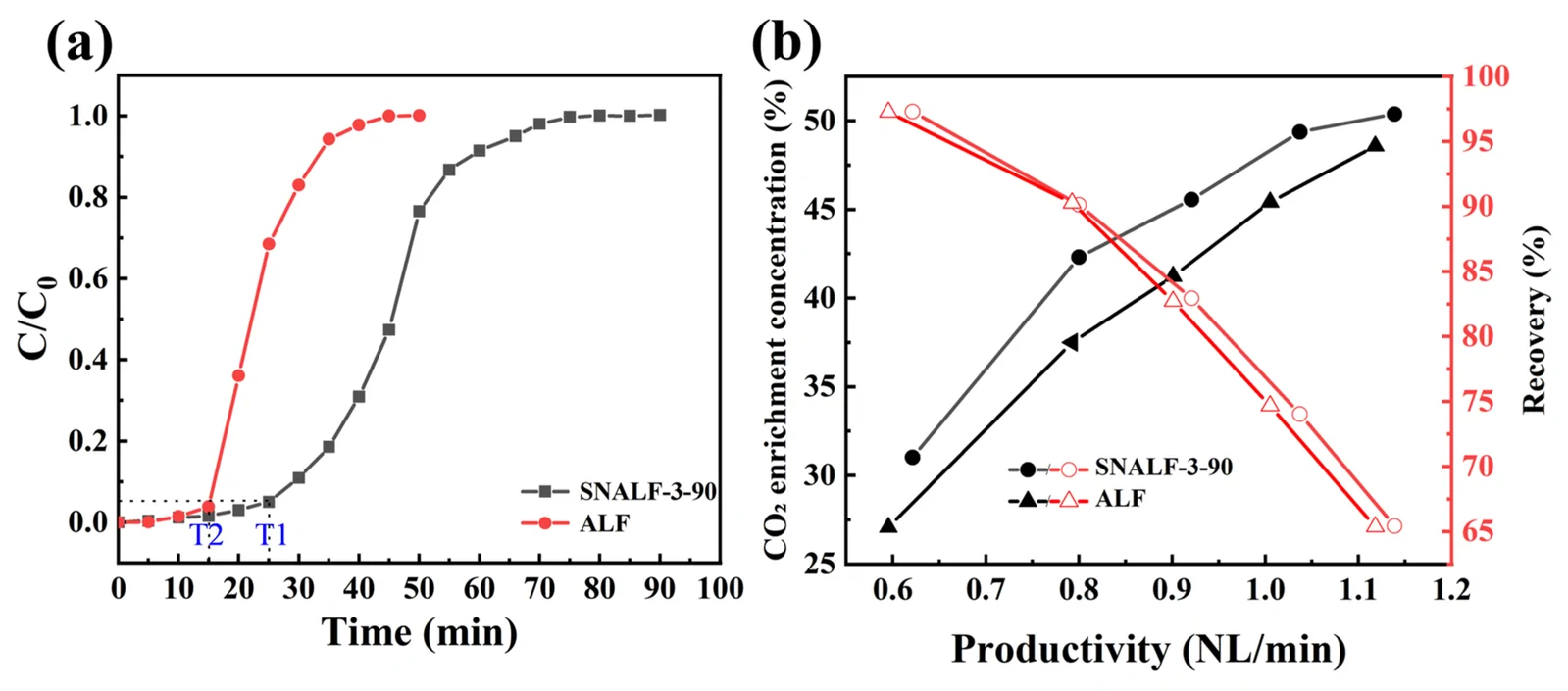
(**a**) Breakthrough curves of 6.5/11/82.5 O_2_/CO_2_/N_2_ mixture with ALF and SNALF-3-90 on the PSA unit; (**b**) the relationship among productivity, CO_2_ enrichment concentration, and recovery in PSA experiment of SNALF-3-90 and ALF.

The breakthrough curves of pristine ALF and optimized SNALF-3-90 in simulated flue gas are measured using the PSA, with the results presented in Figure 10a. SNALF-3-90 exhibits a longer breakthrough time (T1 > T2) and higher CO_2_ adsorption capacity compared to ALF. We further tune the feed gas flow rate (productivity) in the PSA cycle to explore the mutual correlations among productivity, CO_2_ enrichment concentration, and recovery, with the results presented in Figure 10b. Comparative analysis of the three key metrics confirms that SNALF-3-90 possesses outstanding flue gas CO_2_ separation capability. Specifically, the CO_2_ concentration could be enriched from 11% to over 42%, with a CO_2_ recovery of 90.13% and a productivity of 0.80 NL/min. Interestingly, a trade-off relationship exists among the three parameters—productivity, CO_2_ enrichment concentration, and recovery. Increasing productivity leads to a reduction in recovery, while enhancing recovery simultaneously decreases productivity and CO_2_ enrichment concentration. Therefore, optimal operating parameters should be selected in accordance with practical industrial requirements.

### 2.5. DFT Calculations

All density functional theory (DFT) computations were carried out using the Vienna ab initio simulation package (VASP) [44,45]. The exchange–correlation functional was treated with the Perdew–Burke–Ernzerhof (PBE) form within the generalized gradient approximation (GGA) [46]. Electron–ion interactions were handled via the projector augmented wave (PAW) method [47]. A plane-wave cutoff energy of 450 eV was adopted, and the Brillouin zone was sampled using a 2 × 2 × 2 gamma-centered Monkhorst–Pack k-point mesh. The convergence thresholds for energy and atomic forces during self-consistent iterations were set to 10^−5^ eV and 0.05 eV/Å, respectively. Long-range van der Waals (vdW) interactions were taken into account through the DFT-D3 correction scheme [48]. The adsorption energy (*E*_ads_) was determined using Equation (1):

*E*_ads_ = *E*_total_ − *E*_slab_ − *E*_adsorbate_ (1)

Here, *E*_total_ denotes the total electronic energy of the combined adsorbate–slab system; *E*_slab_ represents the electronic energy of the clean slab; and *E*_adsorbate_ corresponds to the electronic energy of the isolated molecule(s). A negative value for *E*_ads_ indicates exothermic adsorption, and the larger the absolute value, the stronger the adsorption.

To construct the defective SNALF model, we started from the crystallographic unit cell of pristine ALF, as determined by XRD, incorporating the role of the citric acid modifier as characterized by ^1^H NMR and TGA. A supercell was then generated, and a single formate ligand (HCOO^−^) was removed from a specific aluminium coordination site, creating a ligand-missing defect. This corresponds to a nominal defect concentration of approximately 3.1 mol% relative to the total number of formate groups in the supercell. The defect was placed in an isolated configuration, with a minimum distance of >10 Å between periodic images, ensuring that any defect–defect interactions were negligible and that the computed adsorption behavior reflected the intrinsic effect of a single vacancy. The atomic positions were fully relaxed until the forces fell below the convergence criterion. This defect model is directly motivated by the experimental observations: XRD confirms retention of the ALF framework, while ^1^H NMR and TGA indicate ligand deficiencies and altered coordination environments. The model therefore provides a realistic representation of the defective structure proposed for SNALF, allowing us to probe the role of formate vacancies as active sites for CO_2_ capture.

**Figure 11:**
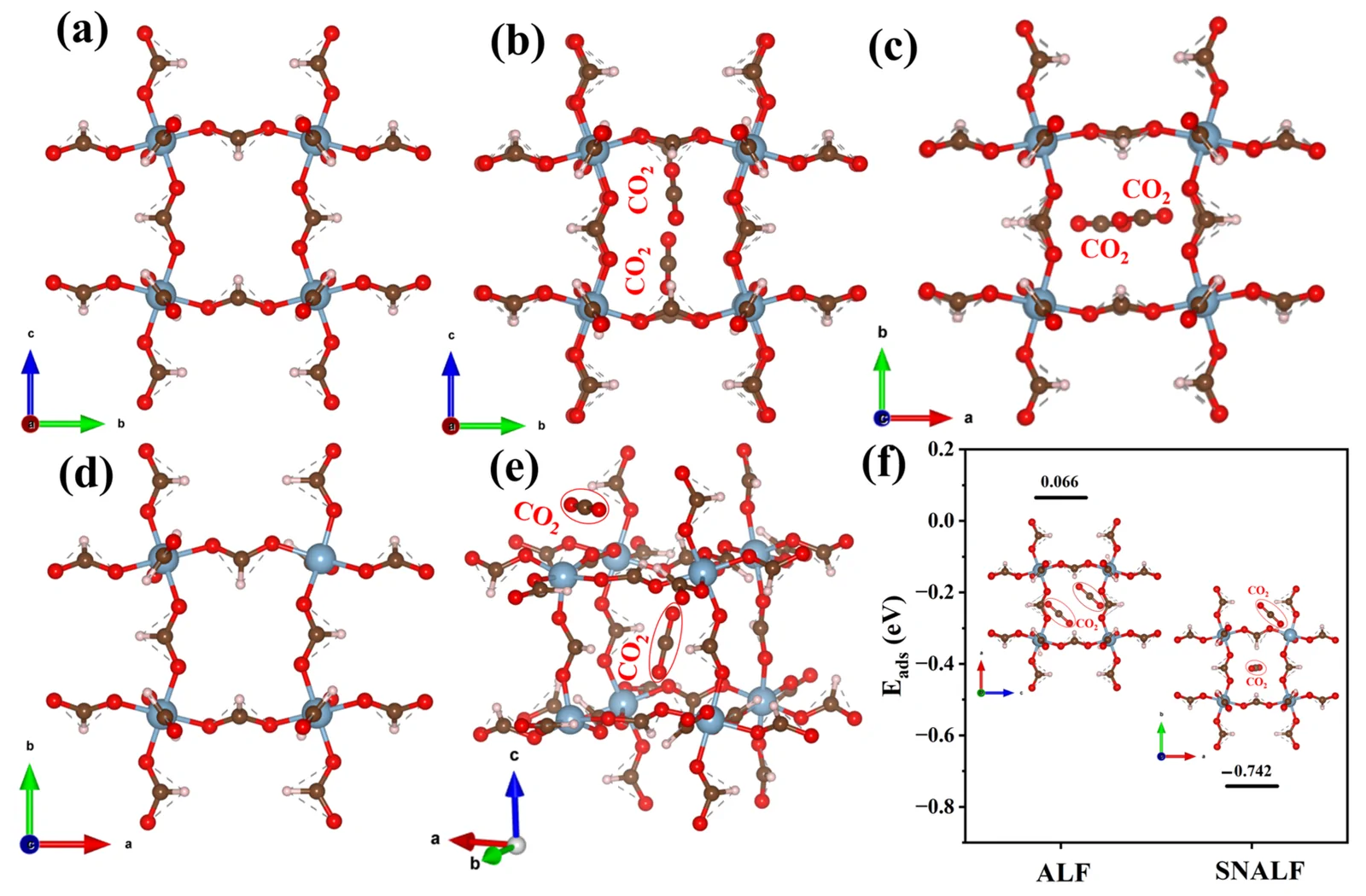
Structures of ALF (**a**–**c**) and SNALF (**d**,**e**) before/upon CO_2_ adsorption; (**f**) adsorption energies of ALF and SNALF calculated by DFT. Color code: red = O, brown = C, white = H, blue = Al.

Figure 11 shows the structures of ALF and ligand-defective SNALF before/upon CO_2_ adsorption. For the pristine ALF surface, the adsorption energy of CO_2_ is only 0.066 eV, showing a slightly positive value. This suggests that the interaction between the surface and CO_2_ is extremely limited, and the adsorption process is thermodynamically unfavorable. This result can be attributed to the lack of effective active sites on the pristine surface, which fails to provide sufficient binding toward CO_2_ molecules. In contrast, the defective SNALF system exhibits distinctly different adsorption behavior. Its adsorption energy decreases significantly to −0.742 eV, demonstrating strong exothermic adsorption characteristics. This value falls within the typical range between physisorption and weak chemisorption, indicating that defect sites can effectively capture CO_2_ molecules and form stable adsorption configurations. The substantial reduction in adsorption energy fully confirms the effectiveness of defect engineering in enhancing surface reactivity. In summary, the introduction of defects substantially modifies the electronic structure and local coordination environment of the SNALF substrate, rendering it an active center for CO_2_ adsorption.

## 3. Materials and Methods

### 3.1. Chemicals

Formic acid (FA) (AR ≥ 98%), Al(OH)_3_ (AR ≥ 99%), ethanol (AR ≥ 99.7%), and citric acid monohydrate (CA) (AR ≥ 99.5%) were supplied by Macklin Biochemical Co., Ltd. Shanghai, China.; nano-sized aluminum hydroxide (nano-Al(OH)_3_) (AR ≥ 99%) with average particle size (10–20 nm) was purchased from Merck & Company, Inc. Darmstadt, Germany.

### 3.2. Synthesis of Adsorbents

**Figure 12:**
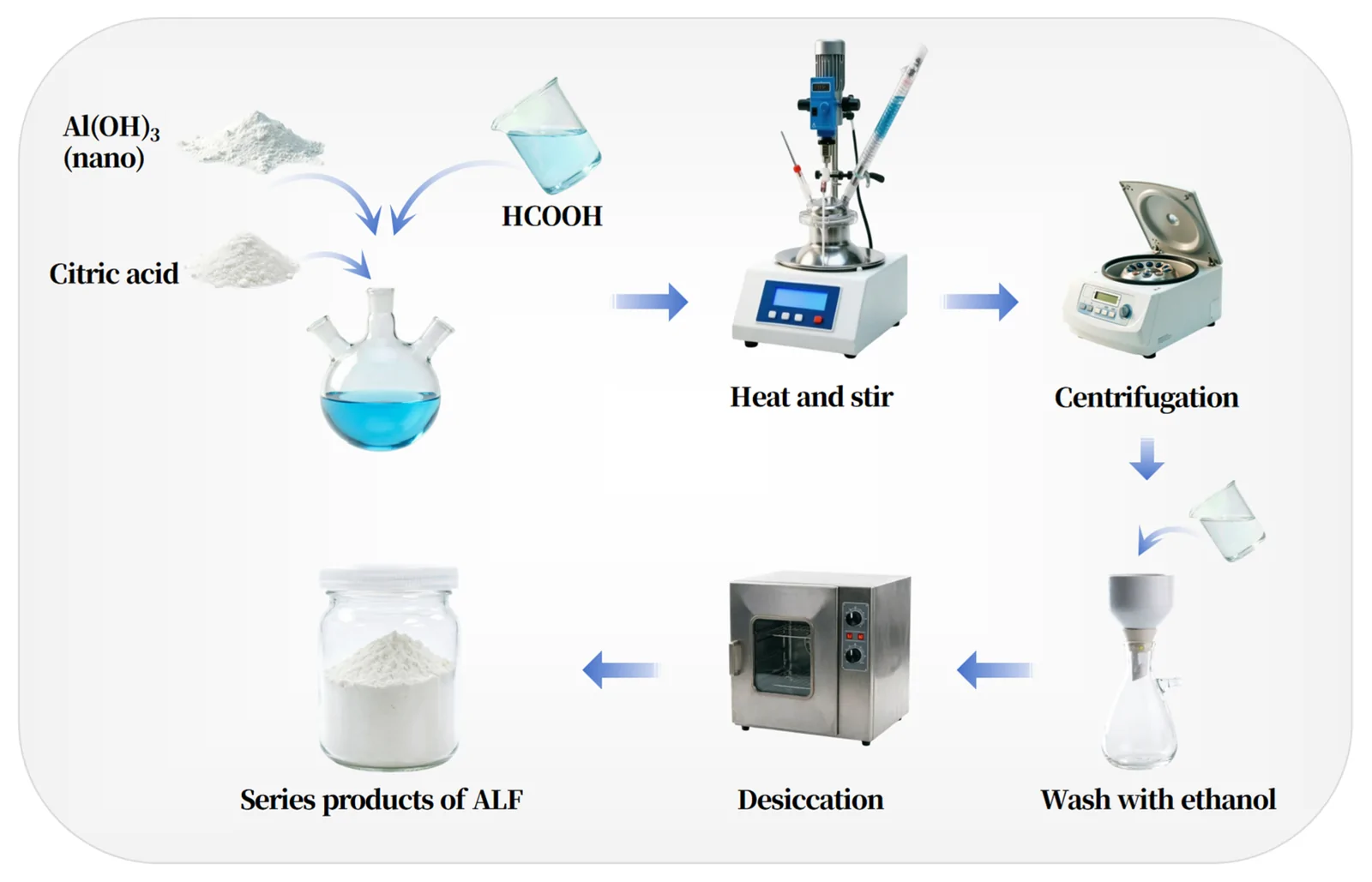
Schematic diagram of the material preparation process (arrows indicate the sequence of operations).

ALF was prepared following a modified literature procedure [21]. In a typical synthesis, Al(OH)_3_ (12 g, 0.15 mol) and formic acid (1000 mL) were refluxed in a 2000 mL three-necked round-bottom flask fitted with a reflux condenser and a magnetic stirrer. The mixture was heated at 90 °C in an oil bath under reflux for 24 h under constant stirring (600 rpm) in air. NALF was synthesized identically, except that nano-Al(OH)_3_ (12 g, 0.15 mol) was used as the aluminum source. SALF was similarly prepared from Al(OH)_3_ (12 g, 0.15 mol) with the addition of CA (4 g, 0.019 mol). For SNALF series (Figure 12), nano-Al(OH)_3_ (12 g, 0.15 mol) and varying dosages of CA were refluxed with formic acid (1000 mL) at different temperatures (see Table 2 for details). For all samples, the resulting solid was isolated by centrifugation (8000 rpm, 10 min), washed with anhydrous ethanol, collected by filtration, and dried at 180 °C for 12 h. The SNALF materials were designated as SNALF-x-y, where x denotes the mass ratio of CA to nano-Al(OH)_3_ and y denotes the reaction temperature. The synthesis was conducted at a laboratory temperature of 25 °C and relative humidity of 70%. Synthesis-detailed, machine-readable synthesis protocols for all materials are provided in the Supporting Information (File S1) as material preparation information files (MPIFs) [49].

### 3.3. Material Characterizations

X-ray diffraction (XRD) patterns were recorded on a Bruker D8 diffractometer using Cu Kα radiation (λ = 1.5406 Å) over a 2θ range of 5–80° at a scanning rate of 5° min^−1^ for crystalline phase identification. Field-emission scanning electron microscopy (FE-SEM) equipped with an energy-dispersive X-ray spectroscopy (EDS) was conducted on an Oxford Ultim Max65 instrument to examine sample morphologies. Fourier-transform infrared (FTIR) spectra were acquired on a Thermo Fisher Nicolet iS200 spectrometer over a wavenumber range of 500–4000 cm^−1^. Thermogravimetric analysis (TGA) was performed on a Mettler Toledo TGA/DSC 3+ simultaneous thermogravimetric analyzer under an argon atmosphere (flow rate: 50 mL min^−1^) from 50 to 600 °C at a heating rate of 5 °C min^−1^ and under an air atmosphere from 50 to 900 °C at a heating rate of 5 °C min^−1^. ^1^H NMR analysis was performed on Quantum-I Plus 600 MHz, using maleic acid as an internal standard and digested in D_2_O containing DCl. CO_2_ adsorption–desorption isotherms were measured at 273 K; prior to measurement, samples were degassed under vacuum at 423 K for 6 h. Pore size distributions (PSDs) were derived from the CO_2_ adsorption isotherms at 273 K using non-local density functional theory (NLDFT). The calculations employed the CO_2_ at 273 K on carbon, slit-shaped pore kernel, which covers pore widths of 0.3–1.5 nm. N_2_ adsorption–desorption isotherms were measured at 77 K on a BSD-660T analyzer (Beishide Instrument, China) using the static volumetric method. samples were degassed under vacuum at 423 K for 6 h; Brunauer–Emmett–Teller (BET) surface areas were calculated from the desorption branch in the relative pressure range *p*/*p*_0_ = 0.0309–0.127. Total pore volumes were taken at *p*/*p*_0_ = 0.990. Average pore diameters were estimated as 4V/A using the BET surface area, and pore size distributions were evaluated from the desorption branch using NLDFT/BJH models as implemented in the instrument software (ASiQwin v3.01).

### 3.4. Adsorption Performance

#### 3.4.1. Equilibrium Adsorption Capacity

An automatic adsorption analyzer (ASAP 2050, Micromeritics Instrument Corporation, Norcross, GA, USA) was employed to determine the equilibrium adsorption capacities of the samples for CO_2_ and N_2_. Prior to the measurements, the samples were degassed under vacuum at 423 K for 6 h. The CO_2_ and N_2_ adsorption isotherms were recorded at 298 K over a pressure range of 0–110 kPa.

#### 3.4.2. Static Adsorption Capacity

**Figure 13:**
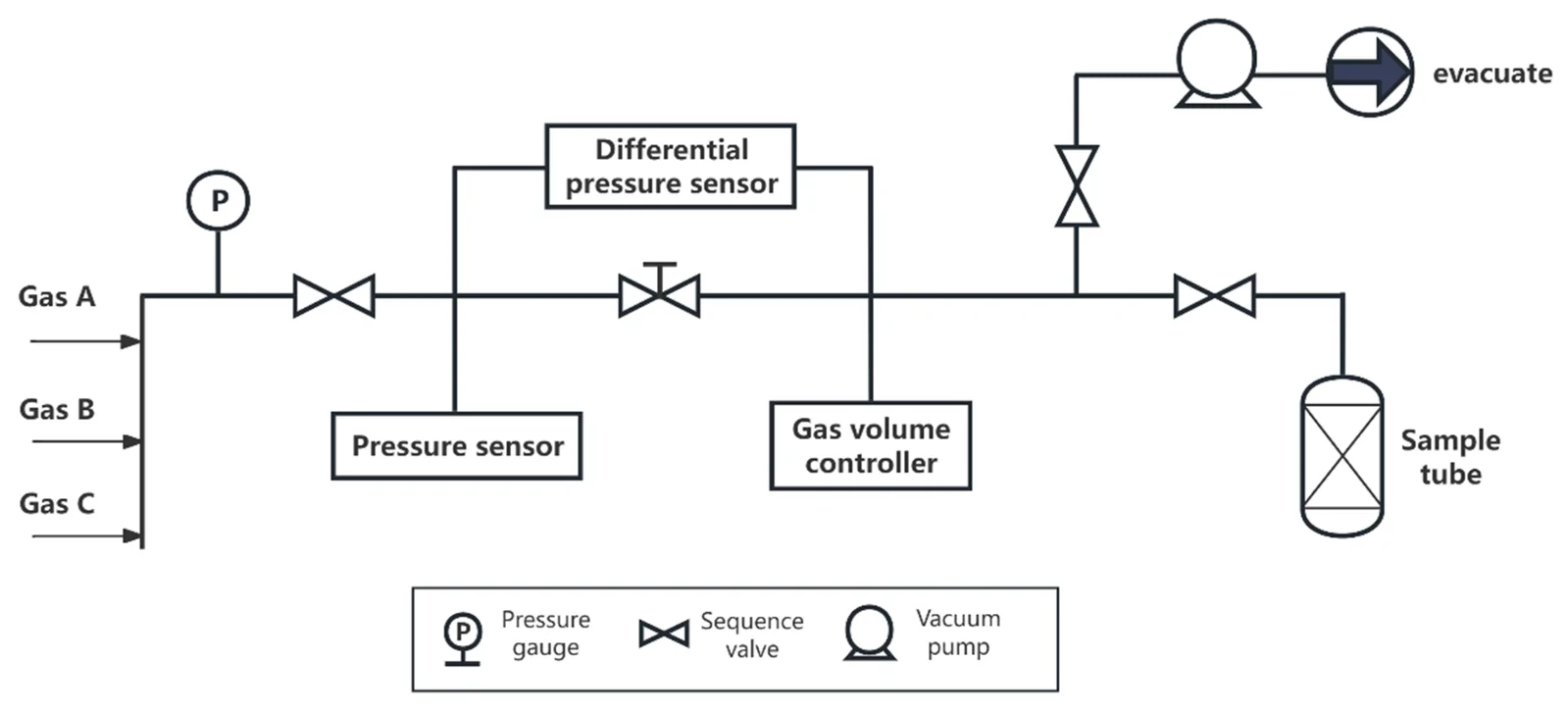
A custom-built manual adsorption device.

A custom-built manual adsorption apparatus was employed to evaluate the CO_2_/N_2_ adsorption performance of the samples (Figure 13). At a specified temperature and atmospheric pressure, the adsorption volumes of He (as a non-adsorbing reference), CO_2_, and N_2_ on the samples were individually measured over a period of 3 min. The adsorption capacity (*q*) and dynamic selectivity(*S*) was calculated according to the following Equations (2) and (3):

(2) $$q_{i}=\frac{(V_{i}-V_{He})K}{22.4\;m}$$

(3) $$S=\frac{q_{{CO}_{2}}}{q_{N_{2}}}$$

where *q_i_* is the adsorption capacity of the adsorbed gas (mmol g^−1^), *V_i_* is the adsorption volume of the adsorbed gas (mL g^−1^), *V_He_* is the adsorption volume of He (mL g^−1^), *K* is the Henry’s constant (mmol g^−1^ kPa^−1^), and *m* is the mass of the adsorbent (g), *S* is dynamic selectivity, and $q_{CO_{2}}$ and $q_{N_{2}}$ represent the adsorption capacities of pure CO_2_ and N_2_ at 3 min, respectively.

#### 3.4.3. PSA Unit

**Figure 14:**
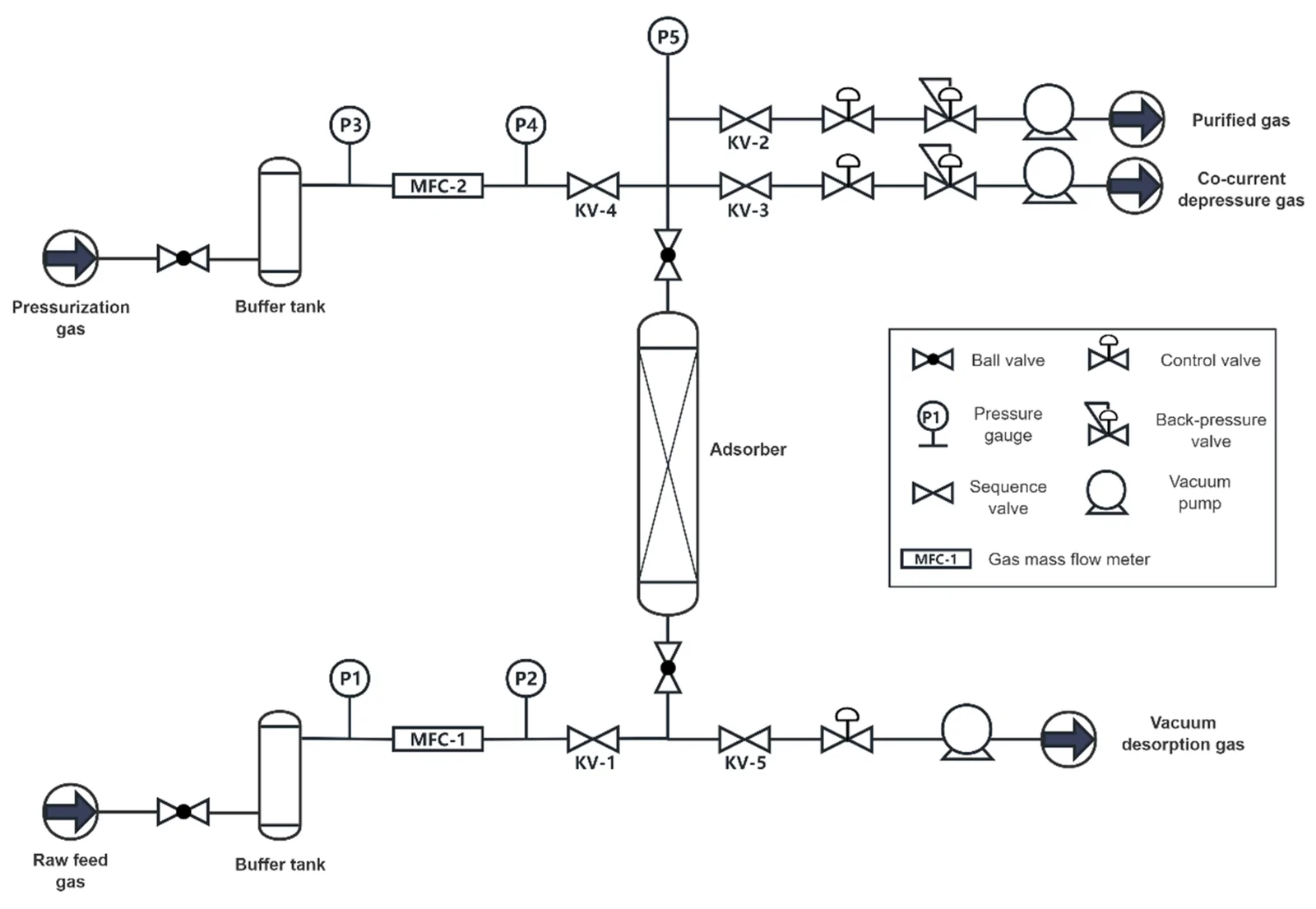
Schematic diagram of PSA experimental device.

The breakthrough experiments were conducted using a PSA unit (Figure 14). The feed gas was simulated flue gas composed of 11 vol.% CO_2_, 6.5 vol.% O_2_, and 82.5 vol.% N_2_. The adsorbent loading was 49.9 g. The adsorption column was first pressurized to 0.2 MPa with pure N_2_; the feed gas was then introduced at a flow rate of 398 mL/min to initiate adsorption. Gas samples were collected at 5 min intervals and analyzed by gas chromatography to obtain the breakthrough curve.

A four-step PSA cycle—comprising pressurization, adsorption, cocurrent depressurization, and vacuum desorption—was investigated for CO_2_ capture from flue gas. Once the process reached steady state, the purified gas, cocurrent depressurization gas, and vacuum desorption gas were collected, quantified, and analyzed by gas chromatography. The CO_2_ enrichment concentration, recovery, and productivity were calculated as shown in Equations (4)–(6):

(4) $$\eta =\frac{C_{1}V_{N1}}{C_{0}(V_{N1}+V_{N2}+V_{N3}-V_{N4})}$$

(5) $$N=\frac{60\times (V_{N1}+V_{N2}+V_{N3}-V_{N4})}{t}$$

(6) $$V_{Ni}=\frac{273.15\times p\times V_{i}}{(273.15+T)\times 760}$$

where *η* is the CO_2_ recovery (%); *N* is the CO_2_ productivity (NL min^−1^); *C*_1_ is the CO_2_ enrichment concentration; *C*_0_ is the CO_2_ concentration in the feed gas; *V*_1_*, V*_2_*, V*_3_*,* and *V*_4_ are the volumes of the vacuum desorption gas, cocurrent depressurization gas, purified gas, and pressurization gas, respectively (mL); *V_N_*_1_, *V_N_*_2_, *V_N_*_3_, and *V_N_*_4_ are the standard-state volumes of the vacuum desorption gas, cocurrent depressurization gas, purified gas, and pressurization gas, respectively (NL); *p* is the ambient measured pressure (mmHg); *T* is the ambient measured temperature (°C); and *t* is the adsorption time (s).

## 4. Conclusions

In summary, this work reports the synthesis of a small-crystallite, ultramicroporous SNALF adsorbent via a dual strategy employing nano-sized aluminum hydroxide as the aluminum source and citric acid as a competitive modulator. The nano-sized aluminum hydroxide accelerates crystal nucleation owing to its high surface energy and small crystallite size. Meanwhile, citric and formic acids compete for aluminum ions, and their removal during washing and drying generates defect sites and reduces crystallite size. This synergistic approach yields a stable framework material with substantially reduced crystallite size and abundant defective active sites. Consequently, SNALF exhibits rapid CO_2_ adsorption kinetics and high capacity. Moreover, both the citric acid dosage and reaction temperature substantially influence the structure and performance of the adsorbent. Under optimized synthesis conditions, the as-synthesized SNALF-3-90 achieved a CO_2_ adsorption capacity of 2.45 mmol g^−1^ within 3 min. PSA tests demonstrated CO_2_ enrichment from 11% to 42% at 0.80 NL min^−1^ productivity, with 90.13% recovery. DFT calculations reveal that pristine ALF barely binds CO_2_ (*E*_ads_ = +0.066 eV), whereas the ligand-defective SNALF surface enables strong exothermic adsorption (*E*_ads_ = −0.742 eV), confirming that the introduced defects act as active centers for CO_2_ capture. This work demonstrates that the synergistic combination of defect engineering and crystallite size reduction substantially enhances the CO_2_ adsorption kinetics of ALF, rendering it a viable candidate for large-scale PSA-based flue gas CO_2_ capture.

## Figures and Tables

**Table 1:** Textural property of prepared materials.

Samples	CO_2_-Accessible Ultramicropore Average Pore Size (nm)	CO_2_-Accessible Ultramicropore Pore Volume(cm^3^/g)
ALF	0.418	0.144
SALF	0.573	0.158
NALF	0.479	0.205
SNALF-3-90	0.479	0.226

**Table 2:** Synthesis conditions of series SNALF samples.

Sample	Mass Ratio (nano-Al(OH)_3_/CA)	Temperature (°C)
SNALF-6-90	6:1	90
SNALF-3-90	3:1	90
SNALF-2-90	2:1	90
SNALF-1.5-90	1.5:1	90
SNALF-3-70	3:1	70
SNALF-3-110	3:1	110

## Data Availability

The data supporting the findings of this study are available within the article and its Supplementary Materials, including the material preparation information files (MPIFs) provided as Files S1. Raw adsorption data are available on request from the corresponding author.

## Supplementary Materials

The following supporting information can be downloaded at: https://www.mdpi.com/article/10.3390/molecules31193500/s1, Figure S1: SEM images of (a) NALF and (c) SNALF-3-90; Particle size distribution of (b) NALF and (d) SNALF-3-90 obtained from SEM image analysis; Figure S2: (a) N_2_ adsorption/desorption isotherms at 77 K and (b) N_2_-derived pore size distributions of ALF, SALF, NALF, and SNALF-3-90; Figure S3: 1HNMR of SNALF-3-90; Figure S4: TGA curves of SNALF-3-90 under air stream; Figure S5: (a)N_2_ adsorption/desorption isotherms at 77 K and (b)N_2_-derived pore size distributions of SNALF-6-90, SNALF-3-90, SNALF-2-90, and SNALF-1.5-90; Table S1: Textural properties of prepared materials; Table S2: Textural properties of prepared materials; File S1: Material preparation information files (MPIF) for synthesized samples.
