# Supplementary material for: A Small-Crystallite Metal–Organic Framework for Efficient CO2 Capture from Flue Gas
Source: Molecules. 2026 Oct 1;31(19):3500. doi: 10.3390/molecules31193500 (PMC13666323; doi:10.3390/molecules31193500)
Supplement: Supplementary file 1 [file molecules-31-03500-s001.zip › Supplementary Materials.pdf]

Supplementary Information

A small-crystallite metal–organic framework for efficient CO_2 capture from flue gas

Ying Yuan [1], Chen Huang [1,*],Yu-jia Chen [1], Xu Li[1], Hong-wei Chen [1], Li-you Liang [1], De-hua Lu [1], Min Liu [1], Ya-min Wu [1], An-min Zhao [1], Yi-hong Wu [2], and Yan-xin Chen [2]

1 State Key Laboratory of Porous Materials for Separation and Conversion, Southwest Research & Design Institute of the Chemical Industry, Chengdu, Sichuan 610225, China

2 State Key Laboratory of Structural Chemistry, Fujian Science & Technology Innovation Laboratory for Optoelectronic Information of China, and Fujian Institute of Research on the Structure of Matter, Chinese Academy of Sciences, Fuzhou, Fujian 361021, China

* Correspondence: huangchen01@sinochem.com; Tel.: (+8613787270942)

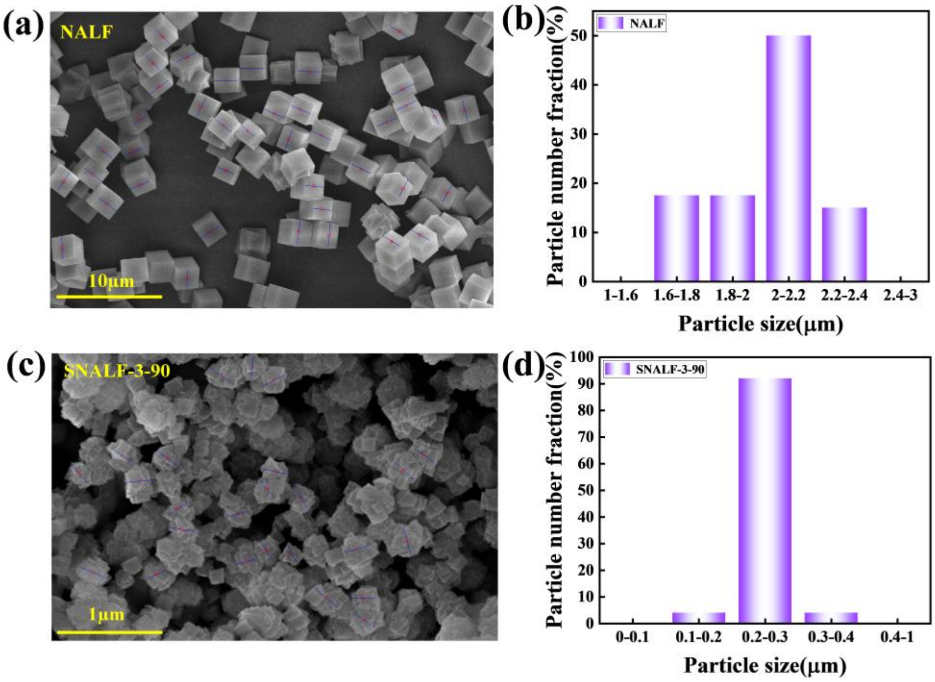

Fig. S1. SEM images of (a) NALF and (c) SNALF-3-90; Particle size distribution of (b) NALF and (d) SNALF-3-90 obtained from SEM image analysis. (Fifty particles were randomly selected from representative SEM images for each sample and measured using Nano Measurer. Particle number fraction (%) was calculated for each size bin. The SEM images in the main text are representative; the histograms provide supporting counting statistics. Reported values are based on n = 50 particles per sample.)

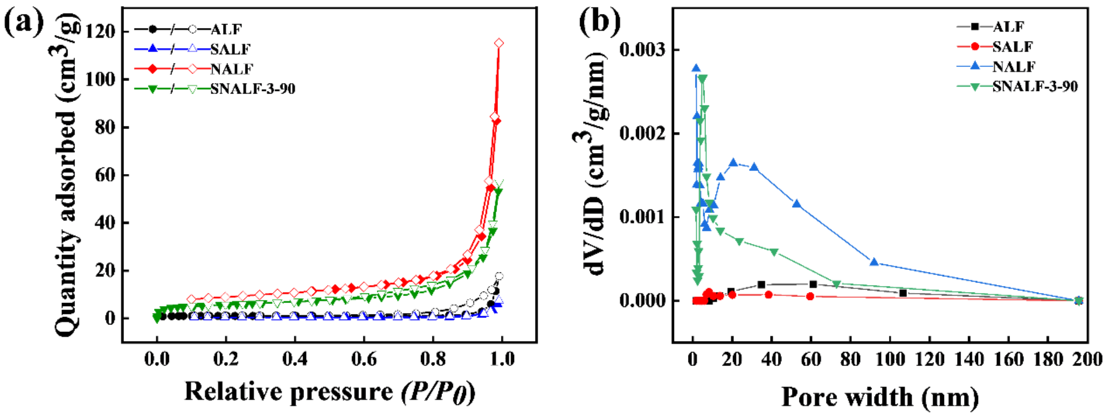

Figure S2. (a)N_2 adsorption/desorption isotherm at 77 K and (b) N_2-derived the pore size distribution of ALF, SALF, NALF and SNALF-3-90.

Table S1. Textural property of prepared materials.

Samples	N_2-BET area (m^2/g)	Average pore size (nm)	Total pore volume(cm^3/g)
ALF	4.1079	24.8302	0.0255
SALF	1.6943	25.9694	0.0110
NALF	32.4871	21.3500	0.1734
SNALF-3-90	20.5566	16.4229	0.0844

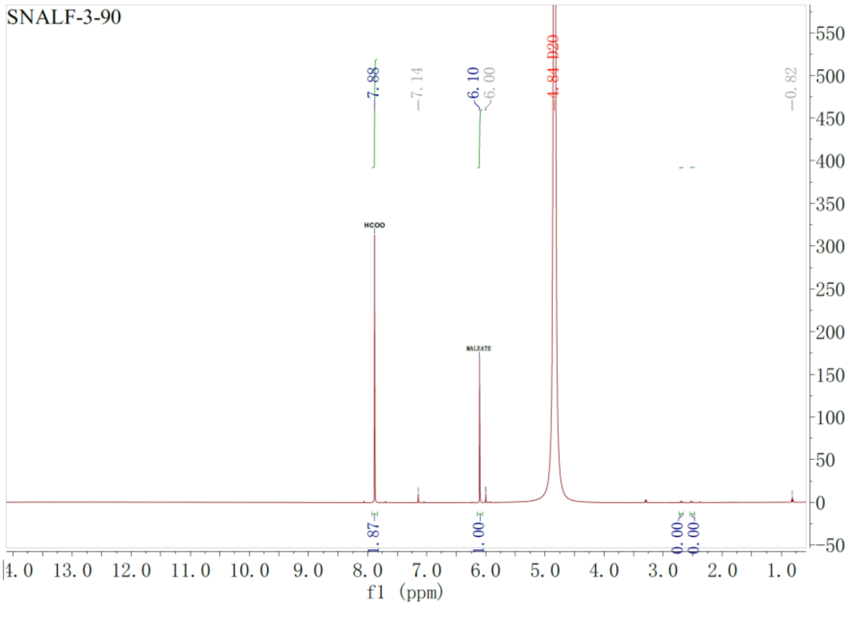

Figure S3. 1HNMR of SNALF-3-90

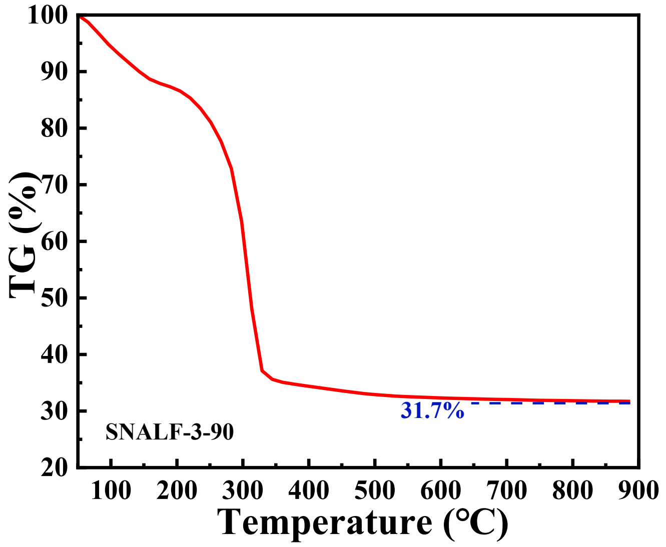

Figure S4. TGA curves of SNALF-3-90 under air stream.

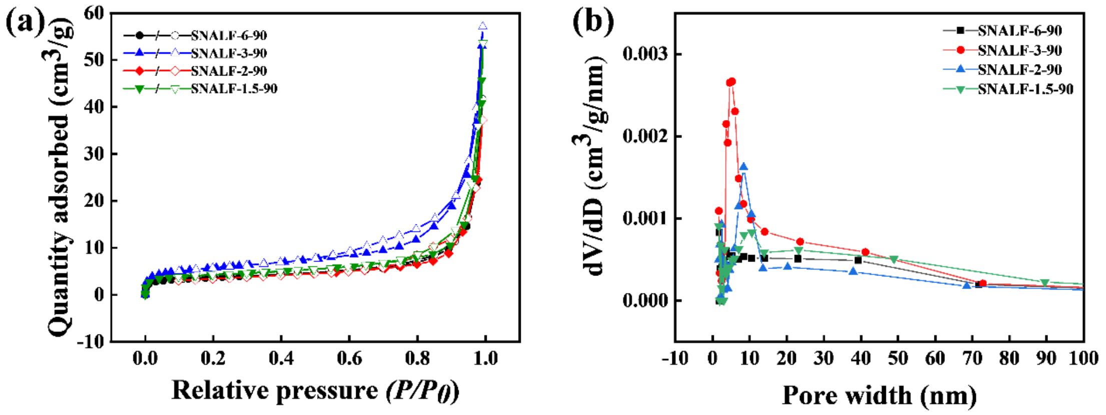

Figure S5. (a)N_2 adsorption/desorption isotherm at 77 K and (b) N_2-derived the pore size distribution of SNALF-6-90, SNALF-3-90, SNALF-2-90 and SNALF-1.5-90.

Table S2. Textural property of prepared materials.

Samples	N_2-BET area (m^2/g)	Average pore size (nm)	Total pore volume(cm^3/g)
SNALF-6-90	13.4359	18.6069	0.0625
SNALF-3-90	20.5566	16.4229	0.0844
SNALF-2-90	12.4167	18.0724	0.0561
SNALF-1.5-90	15.5605	19.2025	0.0747
